# Immunoreactivity of the SARS-CoV-2 entry proteins ACE-2 and TMPRSS-2 in murine models of hormonal manipulation, ageing, and cardiac injury

**DOI:** 10.1038/s41598-021-03181-3

**Published:** 2021-12-14

**Authors:** Susan Bengs, Alexia Rossi, Martina Haberecker, Nidaa Mikail, Alexander Meisel, Achi Haider, Muriel Grämer, Angela Portmann, Atanas Todorov, Christof Schönenberger, Caroline E. Gebhard, Gabriela M. Kuster, Vera Regitz-Zagrosek, Catherine Gebhard

**Affiliations:** 1 Department of Nuclear Medicine, University Hospital Zurich, Zurich, Switzerland; 2 Center for Molecular Cardiology, University of Zurich, Schlieren, Switzerland; 3 Institute of Pathology and Molecular Pathology, University Hospital Zurich, Zurich, Switzerland; 4 Division of Nuclear Medicine and Molecular Imaging, Massachusetts General Hospital, and Department of Radiology, Harvard Medical School, Boston, MA USA; 5 Intensive Care Unit, University Hospital Basel, Basel, Switzerland; 6 Department of Cardiology, University Hospital Basel, Basel, Switzerland; 7 Department of Biomedicine, Myocardial Research, University of Basel, Basel, Switzerland; 8 Charité, Universitätsmedizin Berlin, Berlin, Germany; 9 DZHK (German Centre for Cardiovascular Research), Partner Site Berlin, Berlin, Germany; 10 Division of Cardiology, Department of Internal Medicine II, Medical University of Vienna, Vienna, Austria

**Keywords:** Immunology, Cardiology, Diseases, Medical research, Molecular medicine

## Abstract

Previous work indicates that SARS-CoV-2 virus entry proteins angiotensin-converting enzyme 2 (ACE-2) and the cell surface transmembrane protease serine 2 (TMPRSS-2) are regulated by sex hormones. However, clinical studies addressing this association have yielded conflicting results. We sought to analyze the impact of sex hormones, age, and cardiovascular disease on ACE-2 and TMPRSS-2 expression in different mouse models. ACE-2 and TMPRSS-2 expression was analyzed by immunostaining in a variety of tissues obtained from FVB/N mice undergoing either gonadectomy or sham-surgery and being subjected to ischemia–reperfusion injury or transverse aortic constriction surgery. In lung tissues sex did not have a significant impact on the expression of ACE-2 and TMPRSS-2. On the contrary, following myocardial injury, female sex was associated to a lower expression of ACE-2 at the level of the kidney tubules. In addition, after myocardial injury, a significant correlation between younger age and higher expression of both ACE-2 and TMPRSS-2 was observed for lung alveoli and bronchioli, kidney tubules, and liver sinusoids. Our experimental data indicate that gonadal hormones and biological sex do not alter ACE-2 and TMPRSS-2 expression in the respiratory tract in mice, independent of disease state. Thus, sex differences in ACE-2 and TMPRSS-2 protein expression observed in mice may not explain the higher disease burden of COVID-19 among men.

## Introduction

As of June 2021, the coronavirus disease (COVID)-19 pandemic has caused more than 175 Mio infections and 3.7 Mio deaths worldwide. Pre-existing cardiovascular disease (CVD) has been identified as predominant risk factor for a poor prognosis in COVID-19 patients^1–3^. Suggested mechanisms accounting for this association involve the SARS-CoV-2 entry proteins angiotensin-converting enzyme 2 (ACE-2) and transmembrane protease serine 2 (TMPRSS-2). Emerging data support the hypothesis that the expression of these entry proteins is increased in patients with CVD, particularly in cardiomyocytes^4^. As such, recent results demonstrated that patients with non-ischemic dilated cardiomyopathy, hypertrophic cardiomyopathy, and aortic stenosis exhibit increased expression of ACE-2^5,6^ and that TMPRSS-2 expression increases with age and in males^7^, all of which have been associated with adverse outcomes in COVID-19^3,8,9^. Animal models of gonadectomy showing that estrogen and testosterone exert opposite effects on myocardial ACE-2 expression further support a sexual dimorphism in SARS-CoV-2 entry protein regulation^10^. Despite this, clinical studies addressing the role of sex as well as sex hormones and regulation of ACE-2 and TMPRSS-2 in COVID-19 patients are scarce and the few studies available have yielded conflicting results. While initial reports identified a link between the androgen-mediated phenotype of androgenetic alopecia and COVID-19 disease severity^11,12^ as well as a reduced COVID-19 incidence or disease burden in men receiving anti-androgenic treatment^13–15^, more recent data have refuted this association^16–19^. Also, new results point towards a bivalent role of testosterone in COVID-19 as lower serum testosterone levels predicted poor prognosis and mortality in critically ill men infected with SARS-CoV-2^20,21^. In women, a study from Wuhan, China, demonstrated that higher anti-Müllerian hormone or estradiol levels were associated with a mild COVID-19 disease course in 78 female patients^22^, and a retrospective analysis of 68′466 electronic health records reported a reduction of COVID-19 fatality risk by 50% in 439 postmenopausal women receiving hormone replacement (HR) therapy^23^. Conversely, preliminary data from the UK-based COVID Symptom Study indicate that HR therapy was positively associated with COVID-19 severity^24^, and no increased risk of COVID-19 related mortality was seen in women with gynecologic cancers^25^. Finally, in vitro as well as clinical data showed a reduction of SARS-CoV-2 infection by selective estrogen receptor modulators, but not by agonists/antagonists of estrogen, androgens, or progesterone^26,27^.

Given the paucity of data on ACE-2 and TMPRSS-2 protein expression and their interaction with comorbid health conditions and age, we sought to analyze whether sex hormones, age, and CVD impact ACE-2 and TMPRSS-2 expression in murine models.

## Method

### Study objectives

The ACE-2 receptor is expressed on different types of cells and tissues including human airway epithelia, lung parenchyma, vascular endothelia, renal tubulointerstitial and glomerular cells, central nervous system cells, and small intestinal cells. However, SARS-CoV-2 primarily infects airway epithelial cells. Therefore, the primary objective of our study was to assess whether ACE-2 and TMPRSS-2 expression in lung tissue is regulated by sex, sex hormones, and age at baseline and following myocardial injury. Secondary aims comprised (1) the assessment of ACE-2 and TMPRSS-2 expression in renal and hepatic tissues, and (2) the evaluation of the impact of chronic pressure overload and female reproductive history on ACE-2 and TMPRSS-2 expression in lung, kidney, and liver tissues.

### Mouse models

ACE-2 and TMPRSS-2 expression was analyzed in female and male FVB/N mice (Janvier Labs, France) at different ages (4 months [young], 12 months [middle-aged], and 20–22 months [aged]), with either intact or different hormonal status, as well as different CVD states. All animals were housed in individually ventilated cages with ad libitum access to water and food, and under specific pathogen-free conditions. To test our hypothesis, experimental models of acute myocardial injury (ischemia-reperfusion model) and chronic pressure overload of the left ventricle (LV) as well as evaluation of female mice reproductive history were employed.

#### Acute myocardial injury model

Before surgery, mice were randomly assigned to either gonadectomy (Gx) or sham-surgery at the age of 4 weeks, before reaching sexual maturity. Following gonadectomy, mice were randomized to different experimental groups and 90-days sex HR pellets (in Gx + HR mice) or corresponding vehicle controls (in Gx mice) were implanted. For this purpose, either 0.18 mg of 17β-estradiol or 12.5 mg of testosterone pellets (Innovative Research of America, Sarasota, FL, U.S.) were implanted subcutaneously via a 3 mm incision on the dorsal neck. The pellets were renewed every 90-days under isoflurane anesthesia until final assessment to ensure stable long-term hormone treatment. Subgroups of young and aged mice underwent ischemia–reperfusion injury by inducing transient (30 min) ligation of the left anterior descending artery. Organs were harvested after 24 h of reperfusion. A detailed description of the surgical procedure is reported in the Supplementary Information.

#### Chronic pressure overload model

Young FVB/N mice with intact hormonal status underwent transverse aortic constriction (TAC) or sham surgery to induce persistent pressure overload of the left LV over a total observational time of 28 days. A detailed description of the surgical procedure is reported in the Supplementary Information.

The development of pressure overload of the LV was verified by cardiovascular magnetic resonance imaging on a Bruker BioSpec 70/30 USR magnetic resonance scanner in all mice (data available upon request).

#### Female reproductive history

To assess the potential effect of multiple pregnancies on ACE-2 and TMPRSS-2 expression, tissues of female FVB/N breeders [multiparous] and non-breeders [nulliparous] at the age of 12 months were evaluated.

After harvesting, organs were stored at 4 °C in 4% buffered formaldehyde until further analysis. Blood samples were obtained by cardiac puncture and centrifuged at 10,000*g* for 10 min. The resulting serum samples were snap frozen and stored at − 80 °C until further analysis. All experimental protocols were reviewed and approved by the Commission on Animal Experimentation of the Canton of Zurich and the Cantonal Veterinary Office (link: Application and authorization (admin.ch); license number ZH207/16 and ZH079/18), were carried out in accordance with relevant guidelines and regulations, and reported in accordance with ARRIVE guidelines.

### Serum and tissue analysis

**Figure 1:**
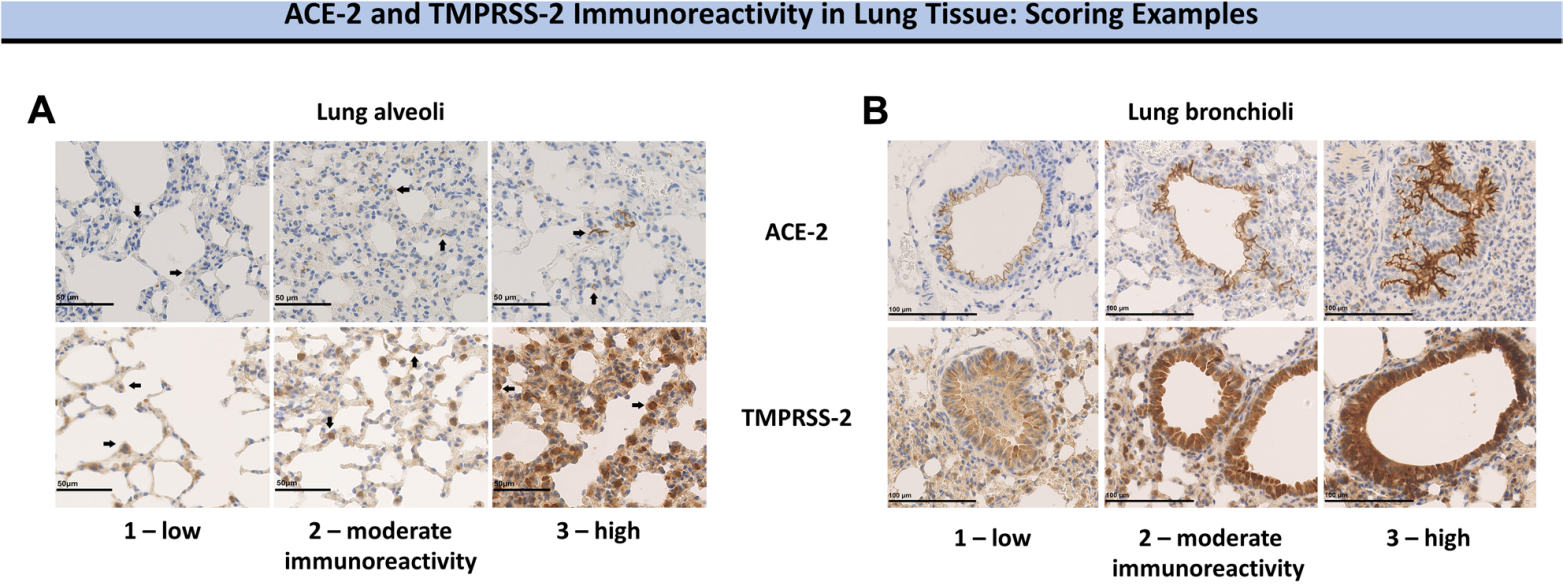
Scoring examples of ACE-2 (upper row) and TMPRSS-2 (lower row) in lung alveoli (**A**) and lung bronchioli (**B**). From left to right: Score = 1 (low immunoreactivity), score = 2 (moderate immunoreactivity), score = 3 (high immunoreactivity). Only membranous staining was evaluated. ACE-2, angiotensin-converting enzyme 2; TMPRSS-2, transmembrane protease serine 2.

Serum sex hormone and soluble ACE-2 (sACE-2) levels were measured using mouse/rat ELISA assays (Calbiotech Inc., El Cajon, CA, U.S and Cloud-Clone Corp., Katy, TX, U.S, respectively) according to the manufacturer’s recommendation. Immunohistochemical stains were performed on formalin-fixed and paraffin embedded tissue using a Bond RX Platform. Immunostainings were performed with established antibodies against the following proteins and visualized using the Bond™ Polymer Refine Detection kit (Leica Biosystems Inc., Buffalo Grove, IL, U.S.): ACE-2; 1:500 dilution (Abcam, #239924) and TMPRSS-2, 1:2,000 dilution (Abcam, #92323). Incubation times were 30 min, respectively, at room temperature. The level of immunoreactivity of ACE-2 and TMPRSS-2 were scored semi-quantitatively (0 = negative; 1 = low; 2 = moderate; 3 = high, Fig. 1A,B). Only membranous staining was evaluated. Additionally, a pattern-based scoring approach was used to evaluate expression levels on liver sinusoids (0 = negative; 1 = portal sinusoids only; 2 = extension to periportal area; 3 = porto-portal/septal pattern). At hepatic level, only the expression of ACE-2 was evaluated since tissue sections were uninterpretable for TMPRSS-2 assessment.

### Statistical analysis

Data are presented as median (interquartile range) for immunostaining and mean ± standard error of the mean (SEM) for serum analyses. For continuous variables prior to analyses, basic assumptions including normal distribution and homogeneity of variances were checked. Unpaired Student’s t-test, Mann–Whitney test, analysis of variance (ANOVA), or Kruskal–Wallis test were used for group comparisons of serum samples. Mann–Whitney test was performed to compare ACE-2 and TMPRSS-2 immunoreactivity between breeders and non-breeders. Ordinal logistic regression was used to evaluate the impact of age, sex, and type of hormonal status on the different levels of expression of ACE-2 and TMPRSS-2 in the different tissues. Statistical tests were two-sided, and significance was set at p < 0.05. Statistical analyses were performed with IBM SPSS statistics v25.0 and R software^28^.

## Results

ACE-2 and TMPRSS-2 expression were assessed in different organs and tissues including lung (alveoli and bronchioli), kidney tubules, and liver sinusoids (Tables 1, 2, 3).

**Table 1:** Impact of age, sex and hormone deprivation/replacement status on ACE-2 and TMPRSS-2 expression at baseline condition.

Tissue	Young mice						Aged mice						Ordinal logistic regression (b coefficient and p-value)			
	Control		Gx		Gx + HR		Control		Gx		Gx + HR					
	Females (n = 9)	Males (n = 9)	Females (n = 10)	Males (n = 10)	Females (n = 10)	Males (n = 10)	Females (n = 10)	Males (n = 10)	Females (n = 10)	Males (n = 10)	Females (n = 7)	Males (n = 7)	Age (ref. aged)	Sex (ref. males)	Gx (ref. control)	Gx + HR (ref. control)
**ACE-expression**																
Lungs																
Alveoli	1.00 (1.00–2.00)	1.00 (1.00–2.00)	1.00 (1.00–2.00)	1.00 (1.00–2.00)	1.00 (1.00–2.00)	1.00 (1.00–2.00)	1.00 (1.00–1.00)	1.00 (1.00–2.00)	1.00 (1.00–1.00)	1.00 (1.00–1.25)	1.00 (1.00–2.00)	1.00 (1.00–2.00)	0.516 (0.225)	− 0.317 (0.450)	− 0.625 (0.228)	− 0.029 (0.954)
Bronchioli	2.00 (2.00–2.00)	2.00 (2.00–2.00)	2.00 (2.00–2.00)	2.00 (2.00–2.00)	2.00 (2.00–2.00)	2.00 (2.00–2.00)	2.00 (1.25–2.00)	2.00 (2.00–2.00)	2.00 (1.25–2.00)	2.00 (1.75–2.00)	2.00 (2.00–2.00)	2.00 (1.00–2.00)	2.766 (**0.010)**	− 0.085 (0.895)	− 0.0115 (0.884)	− 0.399 (0.616)
Kidneys																
Tubules	2.00 (1.00–2.50)	2.00 (2.00–2.25)	2.00 (1.75–3.00)	2.00 (2.00–2.00)	2.00 (1.75–2.25)	2.00 (2.00–3.00)	2.00 (1.00–2.00)	2.00 (1.00–2.00)	2.00 (1.50–2.50)	2.00 (1.00–2.00)	1.00 (1.00–2.00)	2.00 (1.00–3.00)	1.192 **(0.004)**	− 0.425 (0.269)	0.363 (0.430)	0.169 (0.723)
Liver																
Sinusoids	2.00 (2.00–3.00)	2.50 (2.00–3.00)	2.00 (1.75–2.25)	2.00 (2.00–3.00)	2.00 (1.00–2.00)	3.00 (2.00–3.00)	1.00 (1.00–1.25)	1.00 (1.00–1.00)	1.00 (1.00–1.00)	1.00 (1.00–1.25)	1.00 (1.00–1.00)	1.00 (1.00–1.00)	4.225 **(< 0.001)**	− 1.142 **(0.012)**	− 0.332 (0.529)	− 0.369 (0.494)
**TMPRSS-2 expression**																
Lungs																
Alveoli	2.00 (1.00–2.00)	1.00 (1.00–2.00)	1.00 (1.00–2.00)	1.00 (1.00–2.00)	1.50 (1.00–2.00)	1.50 (1.00–2.00)	1.00 (1.00–1.00)	1.00 (1.00–2.00)	1.00 (1.00–1.00)	1.00 (1.00–1.00)	1.00 (1.00–2.00)	1.00 (1.00–1.00)	1.671 **(0.001)**	0.119 (0.790)	− 0.538 (0.330)	− 0.008 (0.988)
Bronchioli	2.00 (2.00–2.00)	2.00 (2.00–2.00)	2.00 (1.00–2.00)	2.00 (1.00–2.00)	2.00 (1.00–2.00)	2.00 (2.00–2.00)	1.00 (1.00–1.25)	1.00 (1.00–1.00)	1.00 (1.00–1.00)	1.00 (1.00–1.00)	1.00 (1.00–2.00)	1.00 (1.00–1.00)	3.250 **(< 0.001)**	− 0.235 (0.642)	− 0.996 (0.116)	− 0.340 (0.596)
Kidneys																
Tubules	2.00 (1.00–2.50)	2.00 (2.00–2.00)	2.00 (1.75–2.25)	2.00 (2.00–3.00)	2.00 (1.75–2.00)	2.00 (2.00–2.00)	2.00 (1.75–2.00)	2.50 (2.00–3.00)	2.00 (2.00–2.00)	2.00 (1.00–2.00)	1.00 (1.00–2.00)	2.00 (1.00–2.00)	0.329 (0.396)	− 0.986 **(0.014)**	− 0.442 (0.342)	− 0.853 (0.080)
Liver																
Sinusoids	Uninterpretable tissue sections															

Data are expressed as median (interquartile range). P-values were derived from ordinal logistic regression.

Significant values are in bold. ACE-2, angiotensin-converting enzyme 2; Gx, gonadectomy; HR, hormone replacement; TMPRSS-2, transmembrane protease serine 2.

**Table 2:** Impact of age, sex and hormone deprivation/replacement status on ACE-2 and TMPRSS-2 expression after acute myocardial injury.

Tissue	Young mice						Aged mice						Ordinal logistic regression (b coefficient and p-value)			
	Control		Gx		Gx + HR		Control		Gx		Gx + HR					
	Females (n = 10)	Males (n = 10)	Females (n = 10)	Males (n = 10)	Females (n = 10)	Males (n = 9)	Females (n = 8)	Males (n = 9)	Females (n = 10)	Males (n = 10)	Females (n = 3)	Males (n = 5)	Age (ref. aged)	Sex (ref. males)	Gx (ref. control)	Gx + HR (ref. control)
**ACE-expression**																
Lungs																
Alveoli	2.50 (2.00–3.00)	2.00 (2.00–3.00)	2.50 (2.00–3.00)	3.00 (2.75–3.00)	2.00 (1.00–3.00)	2.00 (2.00–3.00)	2.00 (2.00–2.00)	2.00 (1.50–3.00)	2.00 (1.75–3.00)	2.00 (1.75–3.00)	2.00 (1.00–)	2.00 (2.00–3.00)	0.787 **(0.044)**	− 0.392 (0.300)	0.479 (0.279)	− 0.167 (0.731)
Bronchioli	2.00 (2.00–3.00)	2.00 (2.00–2.25)	3.00 (2.00–3.00)	3.00 (3.00–3.00)	2.00 (2.00–2.00)	2.00 (2.00–3.00)	2.00 (2.00–2.75)	2.00 (1.50–3.00)	2.00 (1.75–3.00)	2.00 (1.00–3.00)	2.00 (2.00–2.00)	2.00 (2.00–3.00)	0.851 **(0.035)**	− 0.305 (0.431)	0.874 (0.055)	− 0.188 (0.706)
Kidneys																
Tubules	2.00 (1.00–2.00)	2.00 (1.00–2.25)	2.00 (2.00–3.00)	2.00 (2.00–3.00)	1.50 (1.00–3.00)	2.00 (2.00–3.00)	1.00 (1.00–2.00)	2.00 (2.00–3.00)	1.00 (1.00–2.00)	1.00 (1.00–2.00)	1.00 (1.00–)	2.00 (2.00–2.50)	1.047 **(0.009)**	− 1.082 **(0.006)**	0.143 (0.747)	0.289 (0.555)
Liver																
Sinusoids	2.00 (2.00–2.00)	2.00 (1.00–2.00)	1.00 (1.00–2.00)	1.50 (1.00–2.25)	1.00 (1.00–1.00)	1.50 (1.00–2.25)	1.00 (1.00–1.00)	1.00 (1.00–1.00)	1.00 (0.00–1.00)	1.00 (0.75–1.00)	0.00 (0.00–0.00)	1.00 (1.00–2.00)	3.667 **(< 0.001)**	− 0.839 (0.054)	− 1.082 **(0.039)**	− 1.383 **(0.014)**
**TMPRSS-2 expression**																
Lungs																
Alveoli	2.00 (2.00–2.25)	2.00 (1.75–3.00)	2.00 (2.00–2.25)	2.00 (1.75–2.00)	2.00 (2.00–3.00)	2.00 (2.00–3.00)	2.00 (1.00–2.00)	2.00 (1.00–2.00)	1.50 (1.00–2.00)	2.00 (1.75–2.00)	2.00 (2.00–)	2.00 (1.50–2.00)	1.836 **(< 0.001)**	− 0.039 (0.924)	− 0.417 (0.393)	1.062 (0.054)
Bronchioli	2.00 (1.75–2.25)	2.00 (2.00–3.00)	2.00 (1.75–2.00)	2.00 (1.75–2.00)	2.00 (2.00–2.00)	2.00 (2.00–3.00)	2.00 (1.00–2.00)	2.00 (1.00–2.00)	2.00 (1.00–2.25)	2.00 (1.00–2.00)	2.00 (2.00–2.00)	2.00 (1.00–2.00)	1.054 **(0.014)**	− 0.287 (0.473)	− 0.229 (0.621)	0.373 (0.475)
Kidneys																
Tubules	2.00 (1.75–2.00)	2.00 (1.75–3.00)	2.00 (2.00–2.25)	2.00 (2.00–3.00)	2.00 (2.00–3.00)	2.00 (2.00–3.00)	2.00 (1.00–2.00)	2.00 (2.00–2.00)	2.00 (1.00–2.00)	2.00 (1.00–2.00)	2.00 (2.00–2.00)	2.00 (1.50–2.00)	1.287 **(0.005)**	− 0.358 (0.381)	0.415 (0.385)	0.516 (0.330)
Liver																
Sinusoids	Uninterpretable tissue sections															

Data are expressed as median (interquartile range). P-values were derived from ordinal logistic regression.

Significant values are in bold. ACE-2, angiotensin-converting enzyme 2; Gx, gonadectomy; HR, hormone replacement; TMPRSS-2, transmembrane protease serine 2.

**Table 3:** Impact of female reproductive history on ACE-2 and TMPRSS-2 expression.

Tissue	Nulliparous (n = 8)	Multiparous (n = 10)	p-values
**ACE-2 expression**			
Lungs			
Alveoli	1.00 (1.00–1.00)	1.00 (1.00–1.00)	1.000
Bronchioli	2.00 (1.00–2.00)	2.00 (1.00–2.00)	0.796
Kidneys			
Tubules	2.00 (1.00–2.00)	2.00 (2.00–2.00)	0.573
Liver			
Sinusoids	1.00 (1.00–1.00)	1.00 (1.00–1.25)	0.515
**TMPRSS-2 expression**			
Lungs			
Alveoli	1.00 (1.00–1.00)	1.00 (1.00–1.00)	1.000
	1.00 (1.00–1.00)	1.00 (1.00–1.00)	0.439
Kidneys			
Tubules	2.00 (2.00–2.00)	2.00 (1.75–2.00)	0.929
Liver			
Sinusoids	Uninterpretable tissue sections		

Data are expressed as median (interquartile range). P-values were derived from Mann–Whitney test. ACE-2, angiotensin-converting enzyme 2; TMPRSS-2, transmembrane protease serine 2.

### Impact of age, sex, and myocardial injury on ACE-2 and TMPRSS-2 expression in lung tissue

**Figure 2:**
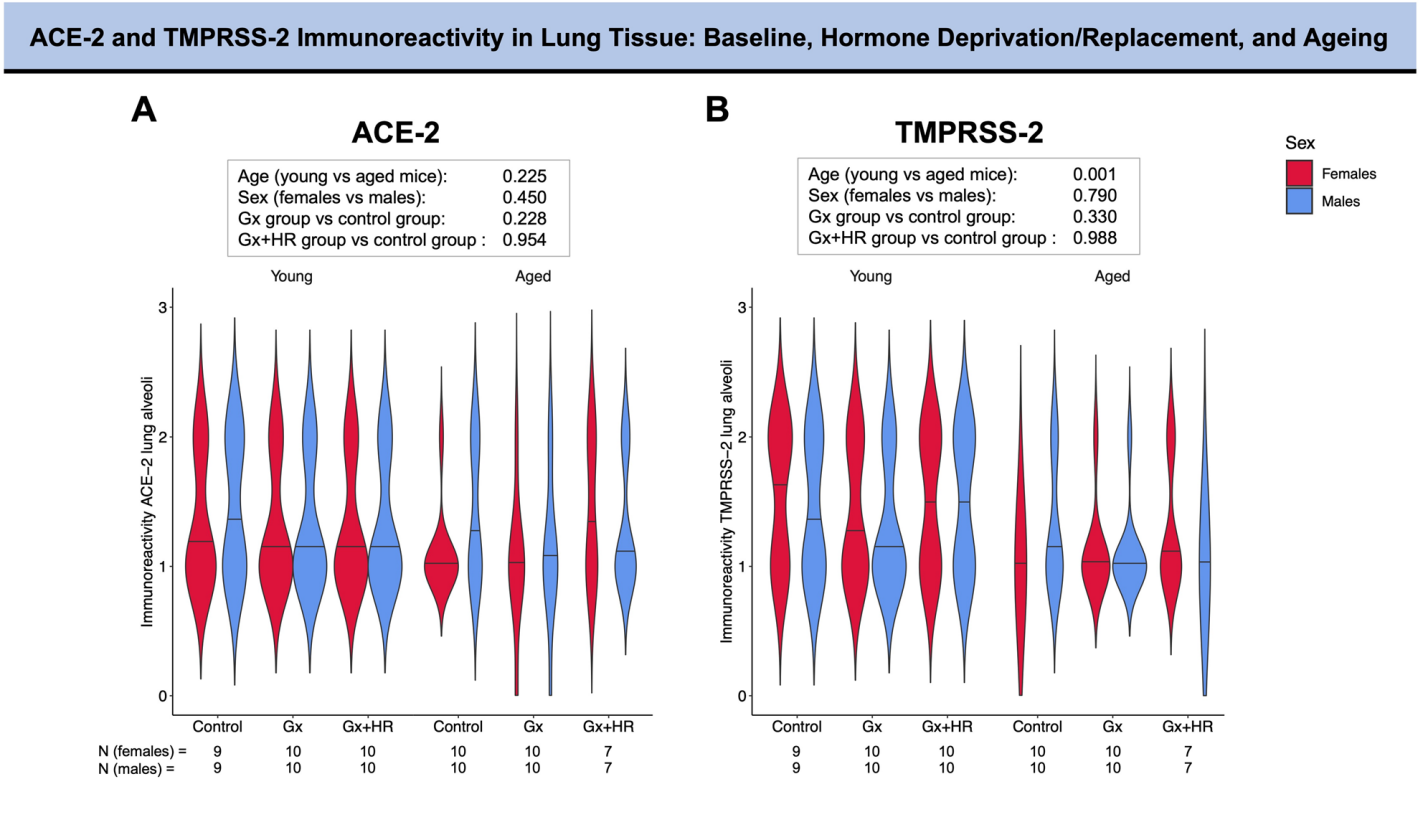
ACE-2 and TMPRSS-2 immunoreactivity in lung alveoli in murine models of ageing and hormonal manipulation. The violin plots provide information on the full data distribution. The horizontal black line indicates the median of the kernel density plot. (**A**) ACE-2 immunoreactivity in young (left panel) and aged (right panel) control animals, gonadectomized animals, and gonadectomized animals receiving long-term hormone replacement. (**B**) TMPRSS-2 immunoreactivity in young (left panel) and aged (right panel) control animals, gonadectomized animals, and gonadectomized animals receiving long-term hormone replacement. Ordinal logistic regression was performed to test the association between the level of expression of ACE-2 and TMPRSS-2 with age, sex, and hormonal status. The resulting p-values are reported in the box. ACE-2, angiotensin-converting enzyme 2; Gx, gonadectomy; HR, hormone replacement; TMPRSS-2, transmembrane protease serine 2.

**Figure 3:**
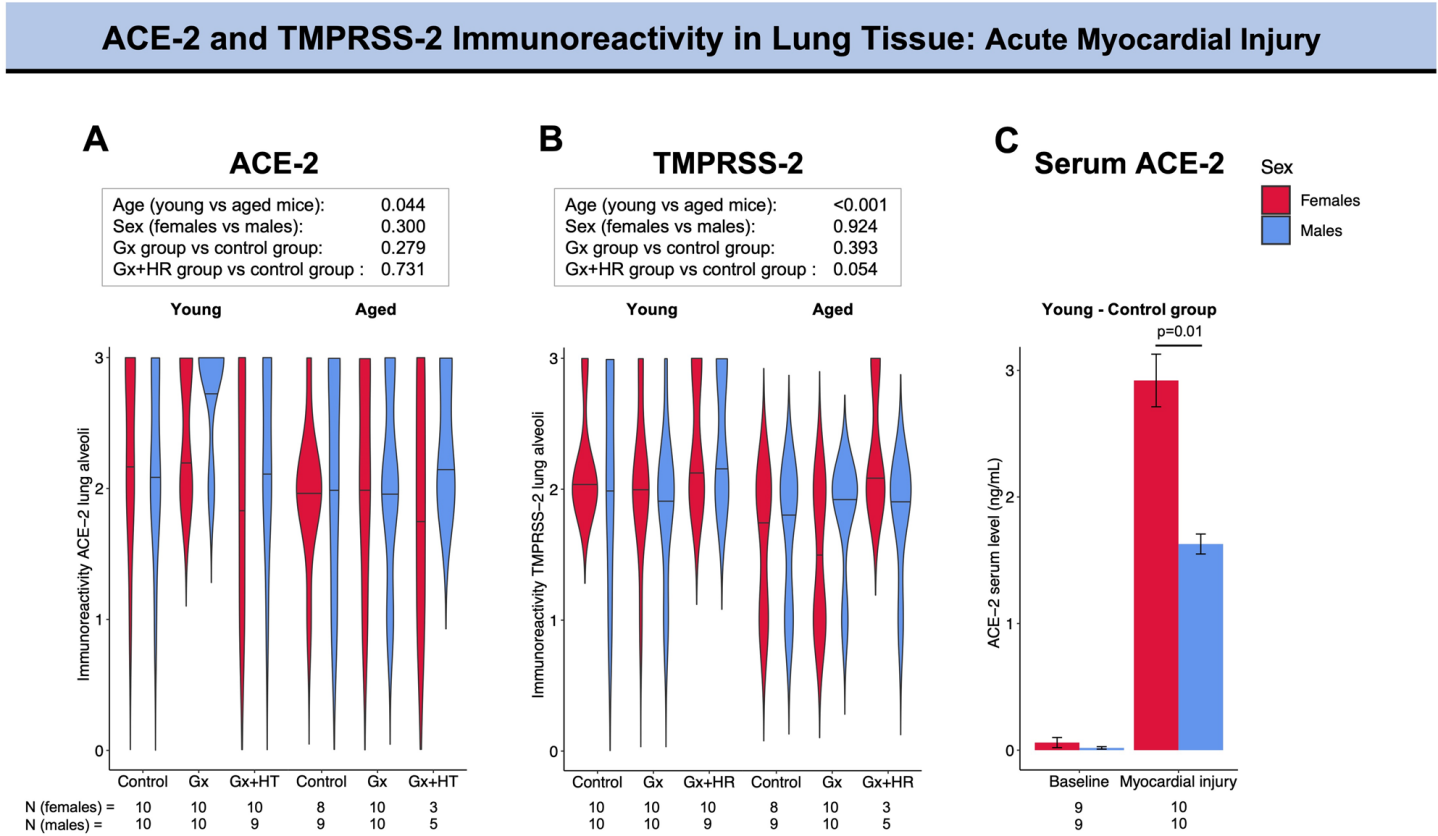
ACE-2 and TMPRSS-2 immunoreactivity in lung alveoli in a murine model of ageing, hormonal manipulation, and myocardial injury. The violin plots provide information on the full data distribution. The horizontal black line indicates the median of the kernel density plot. (**A**) ACE-2 immunoreactivity in young (left panel) and aged (right panel) control animals, gonadectomized animals, and gonadectomized animals receiving long-term hormone replacement following ischemia-reperfusion injury. (**B**) TMPRSS-2 immunoreactivity in young (left panel) and aged (right panel) control animals, gonadectomized animals and gonadectomized animals receiving long-term hormone replacement following ischemia-reperfusion injury. (**C**) Soluble angiotensin-converting enzyme (sACE-2) serum levels in young mice at baseline and following ischemia/reperfusion injury. Ordinal logistic regression was performed to test the association between the level of expression of ACE-2 and TMPRSS-2 with age, sex, and hormonal status. The resulting p-values are reported in the box. Unpaired Student’s t-test was applied for comparison of sACE-2 (only the significant p-value is reported). ACE-2, angiotensin-converting enzyme 2; Gx, gonadectomy; HR, hormone replacement; sACE-2, soluble angiotensin-converting enzyme; TMPRSS-2, transmembrane protease serine 2.

In lung alveoli, sex was not associated with the expression level of ACE-2 and TMPRSS-2 under baseline conditions (p = 0.450 and p = 0.790, respectively) (Fig. 2A,B) and following ischemia–reperfusion injury (p = 0.300 and p = 0.924, respectively) (Fig. 3A,B).

Age did not have a significant impact on the level of ACE-2 expression in the lung alveoli at baseline condition (p = 0.225) (Fig. 2A). On the contrary, higher alveolar ACE-2 expression was associated with younger age following myocardial injury (p = 0.044) (Fig. 3A). Increasing age was associated with a lower expression of pulmonary TMPRSS-2 expression under baseline conditions (p = 0.001) and following myocardial injury (p < 0.001) (Figs. 2B and 3B). Similar observations were found for lung bronchioli (Tables 1 and 2).

Circulating sACE-2 levels were significantly higher in females following ischemia–reperfusion injury as compared to males (p = 0.010, Fig. 3C), while this sex difference was not observed under baseline conditions (p = 0.520).

### Impact of age, sex, and myocardial injury on ACE-2 and TMPRSS-2 expression in kidney, and hepatic tissues

In the kidney tubules, female sex was associated to a lower level of ACE-2 and TMPRSS-2 expression as compared to males following myocardial injury (p = 0.006) and at baseline condition (p = 0.014), respectively. In addition, following myocardial injury, Gx and Gx + HR therapy were associated with a lower extent of ACE-2 expression in the liver sinusoids as compared to the control group (p = 0.039 and p = 0.014, respectively). No additional age, sex, and hormonal status differences in the expression pattern of ACE-2 and TMPRSS-2 were observed in the remaining tissues and experimental groups (Tables 1 and 2).

Younger age was associated with a higher level of ACE-2 expression in kidney tubules and liver sinusoids at baseline (p = 0.004 and p < 0.001, respectively, Table 1) and following myocardial injury (p = 0.009 and p < 0001, respectively, Table 2). Similarly, following myocardial injury, a higher level of expression of TMPRSS-2 in the kidney tubules was correlated with younger age (p = 0.005, Table 2).

### Impact of sex and chronic pressure overload on ACE-2 and TMPRSS-2 expression in lung, kidney, and hepatic tissues

The distribution of ACE-2 and TMPRSS-2 expression level in female and male mice who underwent TAC surgery is reported in the Supplementary Information (Supplemental Table 1 and Supplemental Table 2). Statistical comparisons were not performed due to the small number of animals per group.

### Impact of female reproductive history on ACE-2 and TMPRSS-2 expression

**Figure 4:**
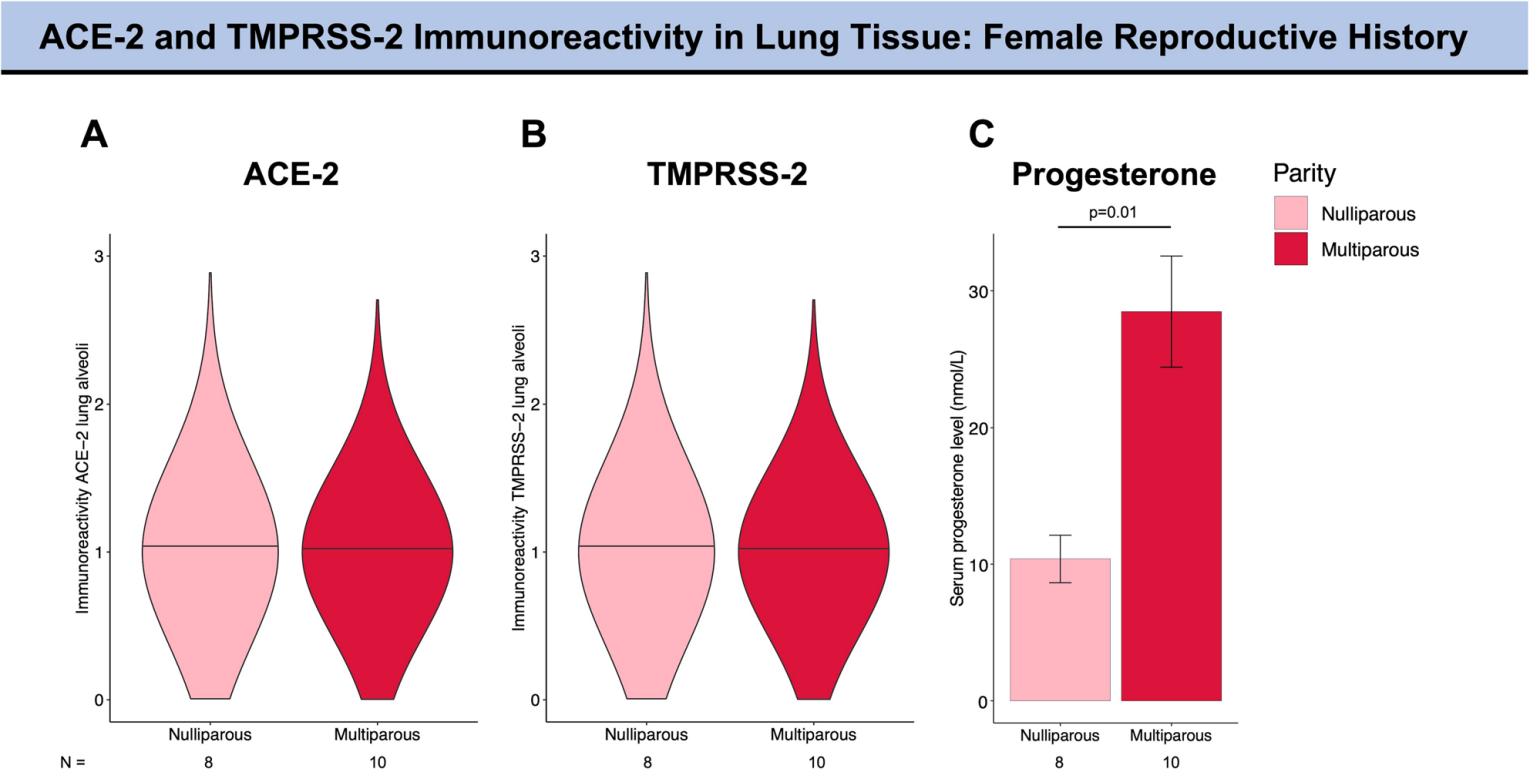
ACE-2 and TMPRSS-2 immunoreactivity in lung alveoli in murine models of pregnancy. The violin plots (**A** and **B**) provide information on the full data distribution. The horizontal black line indicates the median of the kernel density plot. (**A**) ACE-2 immunoreactivity in lung alveoli of 12 months-old multiparous and nulliparous mice. (**B**) TMPRSS-2 immunoreactivity in lung alveoli of 12 months-old multiparous and nulliparous mice. (**C**) Serum progesterone level in both groups. Mann–Whitney test was performed to compare the median of the tissue expression of ACE-2 and TMPRSS-2 between nulliparous and multiparous. Unpaired Student’s t-test was applied for comparison of progesterone levels. Only significant p-values are reported (more details in Table 3). ACE-2, angiotensin-converting enzyme 2; TMPRSS-2, transmembrane protease serine 2.

Female reproductive history did not impact pulmonary expression levels of ACE-2 and TMPRSS-2 as no difference in ACE-2 or TMPRSS-2 expression was observed in multiparous versus nulliparous mice (12 months old; Fig. 4A,B; Table 3). Multiparous mice had significantly higher progesterone levels as compared to nulliparous mice (p = 0.01, Fig. 4C) which is consistent with an increase in progesterone during pregnancy and shortly thereafter.

## Discussion

### Impact of sex on lung expression of ACE-2 and TMPRSS-2

Our experimental data indicate that gonadal hormones and biological sex do not alter the expression of membrane-bound ACE-2 and TMPRSS-2 in the respiratory tract in mice, independent of disease state. Previous data on ACE-2 and TMPRSS-2 expression in lung tissue have yielded conflicting results. Indeed, studies in both human and mouse tissue have reported higher, lower or similar ACE-2 expression in males as compared to females^29–32^ as well as an upregulation (public genomics data) or downregulation (mRNA levels in bronchial epithelial cells) of ACE-2 by estrogen^33,34^. Similarly, while a recent mRNA sequencing study reports a higher TMPRSS-2 expression in men^35^, previous studies did not detect such sex differences across different tissues^31,36^. Species differences, difficulties in precisely measuring membrane-bound ACE-2 expression and activity at the tissue level, confounding variables such as smoking^37^ and obesity^38^ as well as counter-regulatory effects of estrogens on the renin angiotensin system may have caused the high variability of available data.

### Impact of female reproductive history on ACE-2 and TMPRSS-2 expression

Several studies have reported the anti-inflammatory properties of progesterone, including inhibition of neutrophil degranulation and suppression of pro-inflammatory cytokine production^39^. As such, a recent pilot study has shown that subcutaneous administration of progesterone improved clinical status in 42 critically ill men infected with SARS-CoV-2^39^. However, the actions of progesterone on ACE-2 and TMPRSS-2 expression are currently unknown. Multiparous 12-months old mice in our study had significantly higher levels of progesterone than nulliparous mice while estrogen levels were low in both groups. Both ACE-2 and TMPRSS-2 expression did not differ between these two groups across various tissues. Therefore, our data support the hypothesis that the immunomodulatory actions of progesterone^40^ rather than an effect of progesterone on SARS-CoV-2 receptor proteins may play a role in modulating COVID-19 disease course. Nevertheless, further studies addressing COVID-19 morbidity and mortality during pregnancy are needed given that the placenta and the uterus are important sources of ACE-2.

### Impact of sex on ACE-2 and TMPRSS-2 expression in non-cardiac tissues

Pre-existing heart disease has been identified as a strong and independent predictor of adverse outcomes following SARS-CoV-2 infection^1^. Moreover, new cardiac complications are present in up to 12% of hospitalized COVID-19 patients^41^. Previous data suggests that myocardial ACE-2 expression is upregulated in cardiac dysfunction^42^ with two studies reporting more pronounced alterations in males^43,44^. However, the impact of myocardial disease on ACE-2 expression in non-cardiac tissue is currently unknown.

In our study, no sex difference in ACE-2 expression was observed in the experimental groups or organs evaluated with the exception of kidney tubules. This finding is consistent with the reports of two previous studies showing higher ACE-2 activity in male mouse kidneys as compared to females^45,46^.

Overall, the interaction between male sex and CVD in predicting COVID-19 mortality seems independent of sex differences in ACE-2 expression levels. Differences in immune status associated with cardiovascular comorbidities offer potential alternative explanations for the bias towards male deaths in COVID-19.

### Impact of age on ACE-2 and TMPRSS-2 expression

Age is another risk factor for both morbidity and mortality in COVID-19 patients, with children being mostly resistant to the effects of the SARS-CoV-2 virus. In our study, higher pulmonary TMPRSS-2 expression was associated with younger age at baseline condition and following myocardial injury and reperfusion. Similarly, higher TMPRSS-2 expression in kidney tubules was associated with younger age following myocardial injury. Age-dependent changes for alveolar ACE-2 were observed only following myocardial injury. These data contrast with the findings of higher ACE-2 and TMPRSS-2 expression with age reported in a recent study^35^. Species differences as well as differences between protein and gene expression levels may account for these inconsistent findings. Of note, other authors described higher sACE-2 serum levels in children as compared to adults^47^ as well as a decreasing tubulointerstitial ACE-2 expression with age^48^.

## Conclusion

Taken together, sex differences in the expression of ACE-2 and TMPRSS-2 observed in mice may not explain the higher disease burden of COVID-19 among men and contrast with recently reported sex differences on the gene expression level in humans^35^. Further research into the applicability and translational value of preclinical models to study underlying disease mechanisms of COVID-19 is needed.

## Supplementary Information

Supplementary Information.

## Abbreviations

ACE-2: Angiotensin-converting enzyme 2
ANOVA: Analysis of variance
COVID-19: Coronavirus disease
CVD: Cardiovascular disease
Gx: Gonadectomy
HR: Hormone replacement
LV: Left ventricle
sACE-2: Soluble ACE-2
TAC: Transverse aortic constriction
TMPRSS-2: Transmembrane protease serine 2

## References

[1] Bae, S., Kim, S. R., Kim, M. N., Shim, W. J. & Park, S. M. Impact of cardiovascular disease and risk factors on fatal outcomes in patients with COVID-19 according to age: A systematic review and meta-analysis. *Heart* **107**, 373–380. 10.1136/heartjnl-2020-317901 (2021). 33334865 10.1136/heartjnl-2020-317901

[2] Harrison, S. L., Buckley, B. J. R., Rivera-Caravaca, J. M., Zhang, J. & Lip, G. Y. H. Cardiovascular risk factors, cardiovascular disease, and COVID-19: An umbrella review of systematic reviews. *Eur. Heart J. Qual. Care Clin. Outcomes* **7**, 330–339. 10.1093/ehjqcco/qcab029 (2021). 34107535 10.1093/ehjqcco/qcab029 PMC8294691

[3] Williamson, E. J. *et al.* Factors associated with COVID-19-related death using OpenSAFELY. *Nature* **584**, 430–436. 10.1038/s41586-020-2521-4 (2020). 32640463 10.1038/s41586-020-2521-4 PMC7611074

[4] Chung, M. K. *et al.* COVID-19 and cardiovascular disease: From bench to bedside. *Circ. Res.* **128**, 1214–1236. 10.1161/CIRCRESAHA.121.317997 (2021). 33856918 10.1161/CIRCRESAHA.121.317997 PMC8048382

[5] Nicin, L. *et al.* Cell type-specific expression of the putative SARS-CoV-2 receptor ACE2 in human hearts. *Eur. Heart J.* **41**, 1804–1806. 10.1093/eurheartj/ehaa311 (2020). 32293672 10.1093/eurheartj/ehaa311 PMC7184464

[6] Tucker, N. R. *et al.* Myocyte-specific upregulation of ACE2 in cardiovascular disease: Implications for SARS-CoV-2-mediated myocarditis. *Circulation* **142**, 708–710. 10.1161/CIRCULATIONAHA.120.047911 (2020). 32795091 10.1161/CIRCULATIONAHA.120.047911 PMC7424896

[7] Muus, C.*et al.* Integrated analyses of single-cell atlases reveal age, gender, and smoking status associations with cell type-specific expression of mediators of SARS-CoV-2 viral entry and highlights inflammatory programs in putative target cells. *BioRxiv.*10.1101/2020.04.19.049254 (2020).

[8] Gebhard, C., Regitz-Zagrosek, V., Neuhauser, H. K., Morgan, R. & Klein, S. L. Impact of sex and gender on COVID-19 outcomes in Europe. *Biol. Sex Differ.* **11**, 29. 10.1186/s13293-020-00304-9 (2020). 32450906 10.1186/s13293-020-00304-9 PMC7247289

[9] Nguyen, N. T. *et al.* Male gender is a predictor of higher mortality in hospitalized adults with COVID-19. *PLoS ONE* **16**, e0254066. 10.1371/journal.pone.0254066 (2021). 34242273 10.1371/journal.pone.0254066 PMC8270145

[10] Fischer, M., Baessler, A. & Schunkert, H. Renin angiotensin system and gender differences in the cardiovascular system. *Cardiovasc. Res.* **53**, 672–677. 10.1016/s0008-6363(01)00479-5 (2002). 11861038 10.1016/s0008-6363(01)00479-5

[11] Goren, A. *et al.* A preliminary observation: Male pattern hair loss among hospitalized COVID-19 patients in Spain—A potential clue to the role of androgens in COVID-19 severity. *J. Cosmet. Dermatol.* **19**, 1545–1547. 10.1111/jocd.13443 (2020). 32301221 10.1111/jocd.13443

[12] Wambier, C. G. *et al.* Androgenetic alopecia present in the majority of patients hospitalized with COVID-19: The “Gabrin sign”. *J. Am. Acad. Dermatol.* **83**, 680–682. 10.1016/j.jaad.2020.05.079 (2020). 32446821 10.1016/j.jaad.2020.05.079 PMC7242206

[13] Montopoli, M. *et al.* Androgen-deprivation therapies for prostate cancer and risk of infection by SARS-CoV-2: A population-based study (N = 4532). *Ann. Oncol.* **31**, 1040–1045. 10.1016/j.annonc.2020.04.479 (2020). 32387456 10.1016/j.annonc.2020.04.479 PMC7202813

[14] Goren, A. *et al.* Anti-androgens may protect against severe COVID-19 outcomes: Results from a prospective cohort study of 77 hospitalized men. *J. Eur. Acad. Dermatol. Venereol.*10.1111/jdv.16953 (2020). 32977363 10.1111/jdv.16953 PMC7536996

[15] McCoy, J. *et al.* 5-alpha-reductase inhibitors are associated with reduced frequency of COVID-19 symptoms in males with androgenetic alopecia. *J. Eur. Acad. Dermatol. Venereol.*10.1111/jdv.17021 (2020). 33135263 10.1111/jdv.17021

[16] Kwon, D. *et al.* Androgen deprivation therapy and risk of SARS-CoV-2 infection in men with prostate cancer: A University of California (UC) Health System registry study. *J. Clin. Oncol.* **39**, 37 (2021). 10.1016/j.annonc.2021.01.067 PMC7870099 33571636

[17] Tucker, M. D. *et al.* Severe-COVID-19 and mortality among patients (pts) with prostate cancer (PCa) receiving androgen deprivation therapy (ADT). *J. Clin. Oncol.* **39**, 39. 10.1200/JCO.2021.39.6_suppl.39 (2021).

[18] Patel, V. G. *et al.* The role of androgen deprivation therapy on the clinical course of COVID-19 infection in men with prostate cancer. *J. Clin. Oncol.* **39**, 41. 10.1200/JCO.2021.39.6_suppl.41 (2021).

[19] Klein, E. A. *et al.* Androgen deprivation therapy in men with prostate cancer does not affect risk of infection with SARS-CoV-2. *J. Urol.* **205**, 441–443. 10.1097/ju.0000000000001338 (2021). 32897764 10.1097/JU.0000000000001338

[20] Rastrelli, G. *et al.* Low testosterone levels predict clinical adverse outcomes in SARS-CoV-2 pneumonia patients. *Andrology* **9**, 88–98. 10.1111/andr.12821 (2021). 32436355 10.1111/andr.12821 PMC7280645

[21] Rowland, S. P. & O’Brien Bergin, E. Screening for low testosterone is needed for early identification and treatment of men at high risk of mortality from Covid-19. *Crit. Care* **24**, 367. 10.1186/s13054-020-03086-z (2020). 32560707 10.1186/s13054-020-03086-z PMC7303930

[22] Ding, T. *et al.* Potential influence of menstrual status and sex hormones on female SARS-CoV-2 infection: A cross-sectional study from multicentre in Wuhan, China. *Clin. Infect. Dis.*10.1093/cid/ciaa1022 (2020). 32301997 10.1093/cid/ciaa449 PMC7184354

[23] Seeland, U. *et al.* Evidence for treatment with estradiol for women with SARS-CoV-2 infection. *BMC Med.* **18**, 369. 10.1186/s12916-020-01851-z (2020). 33234138 10.1186/s12916-020-01851-z PMC7685778

[24] Costeira, R. *et al.* Estrogen and COVID-19 symptoms: Associations in women from the COVID Symptom Study. *medRxiv*10.1101/2020.07.30.20164921 (2020). 10.1371/journal.pone.0257051 PMC8432854 34506535

[25] Lara, O. D. *et al.* COVID-19 outcomes of patients with gynecologic cancer in New York City. *Cancer* **126**, 4294–4303. 10.1002/cncr.33084 (2020). 32729142 10.1002/cncr.33084 PMC8654115

[26] Mengying, S. *et al.* Sex differences in viral entry protein expression, host responses to SARS-CoV-2, and in vitro responses to sex steroid hormone treatment in COVID-19. *Res. Square*10.21203/rs.3.rs-100914/v1 (2021).

[27] Montopoli, M. *et al.* Clinical outcome of SARS-CoV-2 infection in breast and ovarian cancer patients who underwent antiestrogenic therapy. *Ann. Oncol.*10.1016/j.annonc.2021.01.069 (2021). 33524477 10.1016/j.annonc.2021.01.069 PMC7845554

[28] Team, R. C. *R: A Language and Environment for Statistical Computing. *https://www.R-project.org/ (R Foundation for Statistical Computing, 2021).

[29] Qiao, Y. *et al.* Targeting transcriptional regulation of SARS-CoV-2 entry factors ACE2 and TMPRSS2. *Proc. Natl. Acad. Sci. USA*10.1073/pnas.2021450118 (2020). 33310900 10.1073/pnas.2021450118 PMC7817128

[30] Li, M. Y., Li, L., Zhang, Y. & Wang, X. S. Expression of the SARS-CoV-2 cell receptor gene ACE2 in a wide variety of human tissues. *Infect. Dis. Poverty* **9**, 45. 10.1186/s40249-020-00662-x (2020). 32345362 10.1186/s40249-020-00662-x PMC7186534

[31] Song, H., Seddighzadeh, B., Cooperberg, M. R. & Huang, F. W. Expression of ACE2, the SARS-CoV-2 receptor, and TMPRSS2 in prostate epithelial cells. *Eur. Urol.* **78**, 296–298. 10.1016/j.eururo.2020.04.065 (2020). 32418620 10.1016/j.eururo.2020.04.065 PMC7200365

[32] Baratchian, M. *et al.* Androgen regulation of pulmonary AR, TMPRSS2 and ACE2 with implications for sex-discordant COVID-19 outcomes. *Sci. Rep.* **11**, 11130. 10.1038/s41598-021-90491-1 (2021). 34045511 10.1038/s41598-021-90491-1 PMC8159988

[33] Chen, J. *et al.* Individual variation of the SARS-CoV-2 receptor ACE2 gene expression and regulation. *Aging Cell*10.1111/acel.13168 (2020). 32558150 10.1111/acel.13168 PMC7323071

[34] Stelzig, K. E. *et al.* Estrogen regulates the expression of SARS-CoV-2 receptor ACE2 in differentiated airway epithelial cells. *Am. J. Physiol. Lung Cell Mol. Physiol.* **318**, L1280-l1281. 10.1152/ajplung.00153.2020 (2020). 32432918 10.1152/ajplung.00153.2020 PMC7276982

[35] Muus, C. *et al.* Single-cell meta-analysis of SARS-CoV-2 entry genes across tissues and demographics. *Nat. Med.* **27**, 546–559. 10.1038/s41591-020-01227-z (2021). 33654293 10.1038/s41591-020-01227-z PMC9469728

[36] Baughn, L. B. *et al.* Targeting TMPRSS2 in SARS-CoV-2 Infection. *Mayo Clin. Proc.* **95**, 1989–1999. 10.1016/j.mayocp.2020.06.018 (2020). 32861340 10.1016/j.mayocp.2020.06.018 PMC7368885

[37] Chakladar, J. *et al.* Smoking-mediated upregulation of the androgen pathway leads to increased SARS-CoV-2 susceptibility. *Int. J. Mol. Sci.* **21**, 3627. 10.3390/ijms21103627 (2020). 32455539 10.3390/ijms21103627 PMC7279323

[38] Sarver, D. C. & Wong, G. W. Obesity alters Ace2 and Tmprss2 expression in lung, trachea, and esophagus in a sex-dependent manner: Implications for COVID-19. *Biochem. Biophys. Res. Commun.*10.1016/j.bbrc.2020.10.066 (2020). 33168188 10.1016/j.bbrc.2020.10.066 PMC7605802

[39] Ghandehari, S. *et al.* Progesterone in addition to standard of care versus standard of care alone in the treatment of men hospitalized with moderate to severe COVID-19: A randomized, controlled pilot trial. *Chest*10.1016/j.chest.2021.02.024 (2021). 33621601 10.1016/j.chest.2021.02.024 PMC7896492

[40] Jakovac, H. Sex differences in COVID-19 course and outcome: Progesterone should not be neglected. *J. Appl. Physiol.* **1985**(129), 1007–1008. 10.1152/japplphysiol.00740.2020 (2020). 10.1152/japplphysiol.00740.2020 PMC7607498 33096966

[41] Linschoten, M. *et al.* Cardiac complications in patients hospitalised with COVID-19. *Eur. Heart J. Acute Cardiovasc. Care* **9**, 817–823. 10.1177/2048872620974605 (2020). 33222494 10.1177/2048872620974605 PMC7734244

[42] Bristow, M. R. *et al.* Dynamic regulation of SARS-Cov-2 binding and cell entry mechanisms in remodeled human ventricular myocardium. *JACC Basic Transl. Sci.* **5**, 871–883. 10.1016/j.jacbts.2020.06.007 (2020). 32838074 10.1016/j.jacbts.2020.06.007 PMC7314447

[43] Dalpiaz, P. L. *et al.* Sex hormones promote opposite effects on ACE and ACE2 activity, hypertrophy and cardiac contractility in spontaneously hypertensive rats. *PLoS ONE* **10**, e0127515. 10.1371/journal.pone.0127515 (2015). 26010093 10.1371/journal.pone.0127515 PMC4444272

[44] Stegbauer, J. *et al.* Proteomic analysis reveals upregulation of ACE2 (angiotensin-converting enzyme 2), the putative SARS-CoV-2 receptor in pressure-but not volume-overloaded human hearts. *Hypertension* **76**, e41–e43. 10.1161/hypertensionaha.120.16261 (2020). 32969280 10.1161/HYPERTENSIONAHA.120.16261

[45] Gupte, M. *et al.* Angiotensin converting enzyme 2 contributes to sex differences in the development of obesity hypertension in C57BL/6 mice. *Arterioscler. Thromb. Vasc. Biol.* **32**, 1392–1399. 10.1161/ATVBAHA.112.248559 (2012). 22460555 10.1161/ATVBAHA.112.248559 PMC3355213

[46] Ji, H. *et al.* Sex-specific modulation of blood pressure and the renin-angiotensin system by ACE (angiotensin-converting enzyme) 2. *Hypertension* **76**, 478–487. 10.1161/hypertensionaha.120.15276 (2020). 32564694 10.1161/HYPERTENSIONAHA.120.15276 PMC7365573

[47] Bénéteau-Burnat, B., Baudin, B., Morgant, G., Baumann, F. C. & Giboudeau, J. Serum angiotensin-converting enzyme in healthy and sarcoidotic children: Comparison with the reference interval for adults. *Clin. Chem.* **36**, 344–346 (1990). 2154343

[48] Maksimowski, N., Williams, V. R. & Scholey, J. W. Kidney ACE2 expression: Implications for chronic kidney disease. *PLoS ONE* **15**, e0241534. 10.1371/journal.pone.0241534 (2020). 33125431 10.1371/journal.pone.0241534 PMC7598523

