# Supplementary material for: Immunoreactivity of the SARS-CoV-2 entry proteins ACE-2 and TMPRSS-2 in murine models of hormonal manipulation, ageing, and cardiac injury
Source: Sci Rep. 2021 Dec 14;11:23993. doi: 10.1038/s41598-021-03181-3 (PMC8671541; doi:10.1038/s41598-021-03181-3)
Supplement: Supplementary file 1 — Supplementary Information. [file 41598_2021_3181_MOESM1_ESM.pdf]

Supplementary Information

Immunoreactivity of the SARS-CoV-2 entry proteins ACE-2 and TMPRSS-2 in murine models of hormonal manipulation, ageing, and cardiac injury

Susan Bengs, PhD[1,2,11], Alexia Rossi, MD, PhD[1,2,11], Martina Haberecker, MD[3], Nidaa Mikail, MD[1,2], Alexander Meisel, MD[1,2], Achi Haider, PhD[4], Muriel Grämer, PhD[1,2], Angela Portmann, MSc[1,2], Atanas Todorov[1,2], Christof Schönenberger[5], Caroline E. Gebhard, MD[5], Gabriela M. Kuster, MD[6,7], Vera Regitz-Zagrosek, MD[8,9,11], Catherine Gebhard, MD, PhD[1,2,10,11]

[1]Department of Nuclear Medicine, University Hospital Zurich, Zurich, Switzerland
[2]Center for Molecular Cardiology, University of Zurich, Schlieren, Switzerland
[3]Institute of Pathology and Molecular Pathology, University Hospital Zurich, Zurich, Switzerland
[4]Division of Nuclear Medicine and Molecular Imaging, Massachusetts General Hospital, and Department of Radiology, Harvard Medical School, Boston, Massachusetts, United States
[5]Intensive Care Unit, University Hospital Basel, Basel, Switzerland
[6]Department of Cardiology, University Hospital Basel, Basel, Switzerland
[7]Department of Biomedicine, Myocardial Research, University of Basel, Basel, Switzerland
[8]Charité, Universitätsmedizin Berlin, Berlin, Germany
[9]DZHK (German Centre for Cardiovascular Research), partner site Berlin, Germany
[10]Department of Internal Medicine II, Division of Cardiology, Medical University of Vienna, Vienna, Austria
[11]These authors contributed equally

Short title: Immunoreactivity of ACE-2 and TMPRSS-2

Address for correspondence:
Catherine Gebhard, MD, PhD
Center for Molecular Cardiology
University of Zurich Zurich
8952 Schlieren, Switzerland
Tel.: +41 44 255 2928, Fax.: +41 44 255 4428
Email: catherine.gebhard@usz.ch

Supplemental Method

Description of Surgical Procedures

Analgesia/anesthesia. All mice that underwent either a ligation of the left anterior descending (LAD) coronary artery or transverse aortic constriction (TAC) surgery received subcutaneously 0.1 mg/kg bodyweight buprenorphine (Temgesic®, Indivior Switzerland AG, Baar, Switzerland) at least 30 min prior to the surgery. Animals were anesthetized with 5% isoflurane in oxygen-enriched air. The anesthetic depth was controlled using respiratory rate and depth, negative pedal withdrawal response, movement, and tail pinch reflex. Throughout the surgical intervention, isoflurane was maintained at approximately 2% and body temperature was controlled using the temperature control system TCAT-2 for animals (Harvard Apparatus™, Holliston, Massachusetts, U.S.).

Ligation of the LAD and subsequent ischemia-reperfusion for the acute myocardial injury model. For the ligation of the LAD, the neck and the left side of the rib cage were shaved and the skin disinfected with 80% ethanol. A midline cervical incision was performed to retract the skin and the underlying muscles, thus exposing the trachea. An endotracheal tube was inserted and the thoracic movements were checked. The mechanical ventilation was applied with a tidal volume of 120-180 µL and 120-150 breaths/min. The target respiration rate was around 110 breaths/min (MidiVent Ventilator; Harvard Apparatus™, Holliston, Massachusetts, U.S.). A left lateral thoracotomy was performed between the 3rd and the 4th rib by dissecting the tissue and muscle. The thorax was carefully opened and the pericardial sac was removed. The ligation of the LAD was performed by using an 8-0 Ethilon suture (Polymed Medical Center AG, Opfikon, Switzerland). In addition, a polyethylene tube was placed to prevent damage to the artery. The chest was covered with a sterile moist cotton swap to prevent its desiccation. After 30 min of ischemia, the LAD occlusion was released and an effective reperfusion was confirmed by visible colour restoration of the ischemic tissue. The incision layers were closed by using a 6-0 Prolene (Polymed Medical Center AG, Opfikon, Switzerland) running suture, and the ribs were adapted. The pneumothorax was reversed by draining the thorax with a syringe and removing the air from the pleural space. The skin of the thorax and the neck were closed using 5-0 and 4-0 Prolene (Polymed Medical Center AG, Opfikon, Switzerland) suture, respectively. The intubation was removed as soon as the animal showed spontaneous respiration. To prevent pain, mice were injected subcutaneously with 0.1 mg/kg buprenorphine within 5-6 hours after surgery and at daytime. Imaging of the animals was performed after 24 hours reperfusion.

TAC surgery for the chronic pressure overload model. The neck and the thorax were depilated, cleaned with sterile water and disinfected using Betaseptic (Mundipharma®, Frankfurt am Main, Germany). The foliodrape protect (Paul Hartmann AG, Heidenheim, Germany) was used to cover the lower body. Local analgesic consisting of a lidocaine:bupivacaine mix (10 mg/kg:5 mg/kg

bodyweight) was used and a midline cervical incision from the supra-sternal notch to the mid-chest was performed. The thyroid was lifted and the pre-tracheal muscles were separated by blunt dissection to uncover the trachea. The supra-clavicular muscles were gently pulled up, and a 3–4 mm upper partial sternotomy was performed. The sternal edges were spread gently and the pre-tracheal muscles, mediastinal fat, as well as the thymus were carefully moved aside to visualize the aortic arch and the carotids. The soft tissue was exposed and a tunnel was prepared under the aortic arch. A 5-0 Polyviolene white BRD (Harvard Apparatus$_{TM}$, Holliston, Massachusetts, U.S.) was used to place the ligation under the aortic arch between the origin of the right innominate and left common carotid arteries. A 27G blunted and bended needle was placed in contact to the aortic arch. The ligation was tied snugly around the needle and the arch to achieve a 0.4 mm diameter narrowing and a consequent 65-70% TAC. After ligation, the needle was gently removed. The sternal edges and the intercostal spaces were adapted and the costo-sternal angle was closed using a 6-0 Prolene (Polymed Medical Center AG, Opfikon, Switzerland) suture. In addition, the skin was closed using a 5-0 Prolene (Polymed Medical Center AG, Opfikon, Switzerland) suture. The sham-surgery was performed by the identical constriction procedure without tying the suture around the aorta. After surgery, all animals were injected with 0.1 mg/kg buprenorphine subcutaneously every 5-6 hours during daytime and with 0.05 mg/mL buprenorphine in drinking water over night for at least for 72 hours.

Supplemental Tables

Supplemental Table 1. ACE-2 expression in a model of pressure overload according to sex and type of surgery.

	Sham surgery		TAC surgery	
Score	Females (n=3)	Males (n=3)	Females (n=7)	Males (n=7)
Lung alveoli				
Negative	0	0	0	0
Low	2	1	0	0
Moderate	1	2	3	4
High	0	0	4	3
Lung bronchioli				
Negative	0	0	0	0
Low	0	0	0	0
Moderate	2	1	4	3
High	1	2	3	4
Kidney tubules				
Negative	0	0	0	0
Low	0	0	1	0
Moderate	3	2	1	3
High	0	1	5	4
Liver sinusoids				
Negative	0	0	0	0
Portal sinusoids	1	1	1	0
Extension to periportal area	2	2	4	5
Port-portal / septal pattern	0	0	2	2

Data are expressed as absolute number of observations. ACE-2, angiotensin-converting enzyme 2; TAC, transverse aortic constriction.

Supplemental Table 2. TMPRSS-2 expression in a model of pressure overload according to sex and type of surgery.

	Sham surgery		TAC surgery	
Score	Females (n=3)	Males (n=3)	Females (n=7)	Males (n=7)
Lung alveoli				
Negative	0	0	0	0
Low	1	0	1	2
Moderate	2	3	5	5
High	0	0	1	0
Lung bronchioli				
Negative	0	0	0	0
Low	1	1	2	3
Moderate	2	2	4	4
High	0	0	1	0
Kidney tubules				
Negative	0	0	0	0
Low	2	0	1	1
Moderate	1	3	5	5
High	0	0	1	1
Liver sinusoids				
Uninterpretable tissue sections				

Data are expressed as absolute number of observations. TMPRSS-2, transmembrane protease serine 2; TAC, transverse aortic constriction.
